# Socio-Emotional Skills in Adolescence. Influence of Personal and Extracurricular Variables

**DOI:** 10.3390/ijerph18094811

**Published:** 2021-04-30

**Authors:** Iago Portela-Pino, Myriam Alvariñas-Villaverde, Margarita Pino-Juste

**Affiliations:** 1 Department of Health Sciences, Faculty of Health Sciences, Isabel I University, 09003 Burgos, Spain; iagoportt92@gmail.com; 2 Research Group on Education, Physical Activity and Health (GIES10), Galicia Sur Research Institute (IIS Galicia Sur), SERGAS-UVIGO, 36312 Vigo, Spain; mpino@uvigo.es; 3 Department of Special Didactics, Faculty of Education and Sport Sciences, University of Vigo, 36310 Vigo, Spain; 4 Department of Didactics, School Organization and Research Methods, Faculty of Education and Sport Sciences, University of Vigo, 36310 Vigo, Spain

**Keywords:** soft skills, socio-emotional competences, after-school programmes

## Abstract

Social-emotional skills have been an important object of study in recent years due to their relationship with academic, personal and professional success. The aim of this study was to analyse the relationship between these skills and different influential variables. The participants had a mean age of 14.18 years. The instruments used were the Social Emotional Competence Questionnaire (SECQ) and the Physical Activity Questionnaire for Adolescents (PACQ-A). Generally, the results indicated gender differences and no influence of age. Those who engaged in after-school activities scored higher on social awareness. In addition, artistic and musical extracurricular activities were associated with social-emotional skills, whereas sports activities were not. It was also found that the physical activity index was not related to socioemotional factors, except in self-awareness and in a negative way. It is necessary to analyse the quality of the extracurricular programmes offered and the training of the professionals in charge of their development. It also seems important to take into account the gender perspective in competence work, increasing self-management in girls and relationship management in boys.

## 1. Introduction

Nowadays, it is becoming increasingly important to have sound command of a set of skills that go beyond the traditional technical-professional skills and that are of paramount importance in the labour market and for organisations, referred to as soft skills. Likewise, the role of education in their development is being emphasised [1,2]. They consist of socio-emotional skills that are essential both at work and in personal life and that are associated with success in life [3,4]. They not only help in appropriately comprehending, expressing and regulating one’s own feelings, but also in managing relationships with others, communicating and solving conflicts. Therefore, learning and improving them is crucial to ensure our own well-being and to develop positive relationships with others.

Employers seek employees who have good command of these skills, since they tend to experience greater success in obtaining and sustaining their employment [5]. In fact, Robles [6] reported that technical skills contribute to approximately 15% of career success, while the remaining 85% depends on psycho-emotional or soft skills. He also stated that these skills determine an individual’s strengths to be a good leader, mediator or negotiator. Furthermore, these skills allow for effective teamwork, which is integral to the functioning of many professions [7].

Thus, soft skills contribute to general well-being in the workplace, as they increase stress tolerance and generate pro-social behaviours and a better atmosphere among employees. It is easy to understand that these situations would produce a positive effect on the work-life balance. From a different point of view, it is logical to think that if an individual acquired these skills in their academic or personal life, it would be easier for them to apply them in the workplace.

Various reports [8,9,10] have described these soft skills, also called non-cognitive [11], socio-emotional [12,13] or pervasive skills [2]. They usually refer to a long list of skills that includes personal (optimism, emotion regulation, integrity, responsibility, creativity, initiative, empathy, time management, etc.) and interpersonal skills (problem solving, adaptability, respect, resilience, teamwork, negotiation, mediation, verbal, written and digital communication, active listening, etc.).

Soft skills are transversal competences that are present in both formal and informal situations. One of the most widespread approaches uses a humanistic perspective, going beyond the strictly professional sphere. On one hand, these skills would allow communities to achieve greater productivity, innovation and development but, on the other hand, they should also allow for more committed, respectful, peaceful, inclusive, and, in short, healthier generations, in the broadest sense of the word.

As stated by Wats and Wats [14], the education system should constitute the backbone for the training of competent human resources who can contribute to the development of any country. Thus, it must be up to date according to the significant changes that are happening in the labour market. Therefore, it seems important to start introducing these socio-emotional skills at early ages in school and community contexts. Their implications for youth development are essential. Coneus and Laucht [15] suggested that the acquisition of such skills at early ages may prevent low academic performance, tobacco and alcohol use and risk behaviours such as delinquency. Delhaye et al. [16] indicated that adolescents with depression or low resilience presented poorer socio-emotional skills.

Consequently, education researchers are showing great interest in the training of this type of skills, High Education contexts being most often involved [12,17,18,19,20]. Increasing attention is being also paid to this topic in Primary and Secondary Education. The need for a society made of critical individuals, who are efficient at work and active contributors to social change is clear to education institutions. Besides, a positive relationship between these socio-emotional variables and academic performance has been proved [21,22,23,24].

Programmes to improve social and emotional learning (SEL) skills at school addressed areas such as self-awareness, social awareness, self-management, relationship skills and responsible decision-making [25,26]. These programmes have proved to have significant impact on children and adolescents’ attitudes and behaviours. Various school-based interventions have produced improvements in pro-social skills, problem solving, socio-emotional management and resilience, well-being and even academic performance [27,28,29,30]. A recent update that compared several meta-analyses concluded that universal school-based SEL programmes produced benefits for participating students on both academic and behavioural outcomes and that they persisted along time [31].

Similarly, after-school programmes (ASPs) have included the promotion of these personal and social skills in their aims. The meta-analysis conducted by Durlak et al. [32] revealed that this type of intervention produced a significant enhancement in variables such as self-perception, positive social behaviours, academic achievement, as well as a reduction in problem behaviours. The authors emphasised that ASPs can constitute a privileged community environment for youth personal and social growth. Besides, Shcheglova [33] reported benefits in inter-personal skills and teamwork for individuals involved in extracurricular activities compared to those who were not.

Within extracurricular activities (those performed after school hours), there are numerous studies involving a wide range of categories, such as religious activities, volunteering, visual arts, performing arts, music or sport [34,35,36,37,38,39]. Different outcomes have been observed depending on the type of activity and its effect on soft skills. For example, there is evidence of the benefits of after-school sport on a few of these skills [37,40,41,42]. However, there is also evidence of the lack of influence [38]. Besides, a positive relationship was confirmed between physical activity level and some emotional variables [43,44,45]. Likewise, participation in arts and crafts and music activities was positively related to socio-emotional skills, such as better adaptive behaviour and emotional regulation or positive emotional states, as proved by Metsäpelto and Pulkkinen [38]. Hansen et al. [36] or Muñoz and Mass [46]. In the university context, de Prada et al. [47] confirmed a positive relationship between the participation in extracurricular activities and the acquisition of teamwork socio-emotional skills.

As regards the influence that age may have on these skills, Schoeps et al. [48] did not find any significant difference between 12-to-13-year-old pre-adolescents and 14-to-15-year-old adolescents. Aguilar et al. [27] observed that Primary Education students scored higher in self-consciousness than Secondary Education students. López-González and Oriol [49] reported differences in emotional competence in favour of the youngest adolescents. Besides, Ferrándiz et al. [50], in a study involving students aged 6 to 18, highlighted that the youngest ones scored higher in the variables analysed. Thus, the older the student, the poorer their self-perceived emotional and social competence.

Lastly, gender was a differentiating aspect with regard to this topic in many of the studies reviewed. However, it was generally distinguishing for a certain dimension, but not for the overall socio-emotional competence. Boys usually score higher in emotion self-regulation [51,52,53]. By contrast, girls tend to achieve higher values in variables related to interpersonal relationships [50,54]. Nevertheless, other studies confirmed that gender did not always have influence on socio-emotional skills [49,55].

After reviewing the scientific literature, the aim of the present study was to analyse the relationships between socio-emotional skills and a set of influencing variables: sex; age; physical activity index; attendance to after-school activities; and type of after-school activities.

The following hypotheses were established:

**Hypothesis** **1** **(H1).** *Girls present better socio-emotional skills.*

**Hypothesis** **2** **(H2).** *Students who perform after-school activities present better socio-emotional skills.*

**Hypothesis** **3** **(H3).** *Students who do after-school sport present better socio-emotional skills than those who perform other types of after-school activities.*

**Hypothesis** **4** **(H4).** *Older students present poorer socio-emotional skills.*

**Hypothesis** **5** **(H5).** *Students with higher physical activity index present better socio-emotional skills.*

## 2. Materials and Methods

### 2.1. Participants

A non-probabilistic and intentional sampling method was used. In the sample 48% were boys (466) and 51.7% were girls (498), with a mean age of 14.18 years (SD = 1.28) and with the minimum age being 11 and the maximum 18. They attend 16 schools in Galicia (Spain). In the school year, 31.4% were in 1st year of Compulsory Secondary Education (ESO), 20.5% in 2nd year of ESO, 35.6% in 3rd year of ESO, and 12.5% in 4th year of ESO.

### 2.2. Instrument

The Social Emotional Competence Questionnaire (SECQ) by Zhou and Ee [13] was used to assess social-emotional skills. It was designed to test whether children and adolescents are aware of themselves and others and how they respond in different contexts: family, school and community. The instrument consisted of five factors organised into 25 items on a Lickert-type scale from 1 to 6. 1 meant strongly disagree with the proposed statement and 6 meant strongly agree. The factors were: self-awareness (items 1 to 5); social awareness (items 6 to 10); self-management (items 11 to 15); relationship management (items 16 to 20); and responsible decision-making (items 21 to 25).

The practice of physical activity was evaluated by means of the Physical Activity Questionnaire for Adolescents (PAQ-A) which recorded the activity carried out in the last 7 days during leisure time, physical education classes, after-school hours, and weekends. In addition, it recorded whether any illness or other event hindered the physical activity or sport practice. The overall result of the test is a score from 1 to 5 points that allows a graduation in the level of physical activity performed [56]. PAQ-A was validated for the Spanish population [57,58].

The personal and academic variables were collected in an ad hoc questionnaire and are: school; geographic location; gender; grade; age; academic record; extracurricular activities; and type of activities.

The students were weighed in their underwear with the SECA mod.701 medical scale (accuracy 0.1kg) and their height was measured with the SECA mod.213 stadiometer (accuracy 1mm). BMI was calculated and the diagnosis of overweight and obesity was established using Cole’s international reference standards [59].

### 2.3. Procedure

The study was conducted in 2019. The questionnaire was collectively administered to the students in different schools from the Autonomous Community of Galicia (Spain) with prior authorization from both the school and families. After communicating the appropriate instructions and once the informed consent form was signed, all students voluntarily and individually completed the requested information in their group-class. The ethical research protocols were fulfilled with a special emphasis on confidentiality.

### 2.4. Statistical Data Analysis

Comparison of means was performed by Student’s test and analysis of variance (ANOVA) followed by the Bonferroni post-hoc test. Correlations were determined using Pearson’s correlation coefficient. A *p*-value < 0.05 was considered statistically significant.

Cronbach’s Alpha reliability coefficient was calculated to estimate the internal consistency of the instrument.

The results were statistically analysed using SPSS software (SPSS, Chicago, IL, USA) considering a relationship to be statistically significant when *p* < 0.05.

As far as alpha values are concerned, all factors exceeded 0.799, with the consistency of the SECQ (α = 0.914) and the PAQ (α = 0.80) being very high (Table 1).

## 3. Results

The young people in the sample had an adequate Body Mass Index (BMI = 20.53) and a moderate to low physical activity index (=2.94). With respect to socioemotional competence, the mean score was medium-high, with self-awareness being the most valued dimension and self-management the least valued. Finally, the values of skewness and kurtosis were within the interval ±2. Consequently, it is estimated that univariate normality was present (Table 2).

The analysis also revealed that 84.2% of pupils take part in extracurricular activities. Almost half of them take part in sporting activities (46.2%). This is followed by academic activities, mainly focused on remedial classes (21.7%), musical activities (10.5%) and artistic activities (5.8%). Only 15.9% do not engage in any extracurricular activities.

With regard to the difference in means according to the gender variable, in the variables relating to socio-emotional competences, it can be seen that there were no differences found in self-awareness, social awareness or decision-making between boys and girls. However, there were significant differences in self-management, which was found to be greater in boys, and in relationship management, which was found to be greater in girls (Table 3).

When we analysed the different factors according to whether the students carry out extracurricular activities, we found that there were no differences with respect to the different socioemotional factors, except for the social awareness factor, which was higher in those students who carry out extracurricular activities (Table 4).

We can investigate this issue in more detail if we consider the type of activities they do (Table 5). It can be seen that the extracurricular activities carried out do not influence self-management, but they do influence other skills. In fact, students who take part in sports activities have lower levels of socioemotional skills. Those who do music and art activities excel in self-awareness, social awareness, relationship management and decision-making. Above all, this difference is established among those who do not do any sport activities. In self-awareness and relationship management, students who do sports activities have a lower mean than those who do not do any activity.

The physical activity index had a relationship, albeit a low one, with age. The lower the index, the older the student. It is also found that the physical activity index was not related to the socioemotional factors, except for self-awareness (in a very low and negative way).

Neither the total SECQ score nor any of the factors of this scale were related to age, except in the case of self-management, which was found to be higher with increasing age. Finally, as can be seen, the relationship between the factors of the scale was significant, positive and with a medium correlation (Table 6).

## 4. Conclusions

The purpose of this study was to determine the relationship between socioemotional skills in adolescents and different influential variables, thus contributing to the improvement of knowledge on this subject.

In the last two decades, the great interest in the world of emotions has become evident, due to their important involvement in all facets of life. Nowadays, their link with learning and socio-personal well-being has been sufficiently proven. SEL programmes improve academic performance, reduce aggressive behaviour and help with anxiety/depression problems [29]. For this reason, in many countries, content has been incorporated into the educational landscape that works especially on these dimensions of the individual. Within this context, there is evidence of studies in both formal [27,28] and non-formal settings [32,33].

One of the most analysed questions in adolescent students corresponds to knowledge of the differences in socioemotional skills as a function of the sex variable, or in other words, whether gender is an influential factor in this type of learning. In the present study, girls were found to have greater relationship management skills and boys were found to have greater self-management skills. There was no significant difference in the overall SECQ scores and differences were only observed in these dimensions. Therefore, these results do not allow us to confirm the initial hypothesis that girls have a higher level of socioemotional skills (Ho1).

Our findings are in line with several of the studies reviewed, which have in common that boys have greater self-management; that is, they have more ability to manage their own emotions [52,53]. However, girls have higher scores in the interpersonal dimension [50], relationship management [13,51] or social awareness and prosocial behaviour [51,52]. Schoeps et al. [48] observe in a recent study with Spanish adolescents that girls perceive and understand emotions better than boys, although they present greater emotional problems. In contrast to our data, in works such as that of Rahayu and Mustikasari [55], with Asian children aged 10 to 13 years, no inequalities were observed according to this variable. López-González and Oriol [49] also found no differences in emotional competence between boys and girls in the first cycle of ESO; however, they did find differences in the second cycle, but in favour of boys. These authors argue that their results may be due to the fact that they used a self-report and that it would be ideal to complement it with performance measures.

The second hypothesis of the study (Ho2) suggested that students who engage in extracurricular activities have better social-emotional skills. The results revealed that, overall, this variable has no influence on social-emotional skills; however, in the social awareness factor, it was found to be greater in students who carry out this type of activities than in those who do not. Therefore, we can say that the hypothesis is partially confirmed. It is possible that the socialisation that occurs when participating in this type of activity has an influence on the social awareness of the individual, as they experience situations of relationships between people, where they can understand how others feel, identify with a social group, etc. In line with these findings is the work of Shcheglova [33], who found that those who participate in extracurricular activities obtain more benefits in interpersonal skills and teamwork than those who do not participate.

If we take into account the type of extracurricular activity, it has been found that students who take part in sports activities have a lower level of socioemotional skills. Moreover, in two of the SECQ factors (self-awareness and relationship management) they even have a lower mean than those who do not take part in any activity. This means that the third hypothesis cannot be accepted (Ho3).

The specific field of physical-sports activities is precisely an example of many extracurricular programmes that constitute a potential for learning this type of life skills [41,60]. Thus, improvements have been found in issues such as responsibility or social skills of young people [40,42], in many life effectiveness domains [61] or in various dimensions of psychosocial health, such as better emotional regulation or a lower degree of depressive symptoms, among others [35]. However, our data show just the opposite and are to some extent related to the longitudinal study by Metsäpelto and Pulkkinen [38], which investigates participation in extracurricular activities and their association with socioemotional behaviours. In this case, playing sports (which were the most common extracurricular activities) was found to be associated with social-emotional behaviour.

The explanation we can give for our results relates to the share of negative emotions that sometimes occur in sport-physical contexts: frustrations, competitiveness, tensions, perceived low competence, etc. Hansen et al. [36] found, for example, higher rates of negative peer interaction compared to other types of organised activity. In addition, the approach in extracurricular sports activities may not be adequate; precisely this area is sometimes heavily influenced by the world of sports performance. In order to promote socio-emotional education, real situations of working on values, positive emotions, critical thinking, self-confidence, etc., must be provided. It would be interesting to observe in future research how these classes are developed and what characteristics they have.

On the other hand, we also found that musical and artistic activities were associated with four of the five dimensions of the SECQ. Similarly, Metsäpelto and Pulkkinen [36] concluded that participation in arts and music is related to greater adaptability and work skills. Campayo and Cabedo [46], based on a specific review of the literature on the subject, detail how music is an important tool for the development of emotional competences. Specifically, they explain that music education can be used to express, recognise and better regulate emotions, as well as to work on empathy, cooperation, self-motivation and active listening. Likewise, Hansen et al. [36] found that artistic activities report higher rates of self-knowledge experiences than academic activities and that this type of scenario does not usually involve negative experiences in young people. Chung [62] points out that music involves important social skills such as cooperation, and other studies have suggested that music helps the development of interpersonal skills [63,64].

In the working hypothesis Ho4, it was assumed that older students would have lower levels of social-emotional skills. In this case, the hypothesis cannot be confirmed either, since it has been observed that self-management increases with age. In the rest of the dimensions and in the overall SECQ score, no differences were found.

These data are somewhat similar to those of the study by Aguilar et al. [27], who also used the same instrument with Spanish students. On this occasion, the difference is in the dimension of self-awareness, and no differences were found in the other dimensions of the SECQ. However, this is a comparison between primary and secondary school students. In the study by Schoeps et al. [48], contextualised in ESO, no differences were found according to age. In the study by López-González and Oriol [49], differences in emotional competence were found, with the highest scores corresponding to the group of younger adolescents. Ferrándiz et al. [50], in a study measuring perceived emotional intelligence, also analysed three age groups ranging from 6 to 18 years old. The younger age group is the one that generally presents better values in the dimensions analysed (including stress management and adaptability), so that as age increases, students perceive themselves as less emotionally and socially competent, as these authors explain.

Therefore, as far as the age variable is concerned, we observe that there is no single trend. One possible explanation for our results could be that older students have greater self-management because as they grow older, they join new environments such as gangs or clubs; they interact more and this may imply having to regulate themselves better, increasing their ability to manage their own impulses and emotions.

It has been observed that the rate of physical activity is lower the older the student is. This aspect is common in other research, with the transition from primary to secondary education being particularly important [65,66,67].

Finally, the hypothesis Ho5 postulated that students with a higher physical activity index would have a higher level of socioemotional skills. This hypothesis cannot be confirmed either, since it was found that the physical activity index is not related to the SECQ factors, except for self-awareness and in a negative way. Therefore, people with a higher level of activity have lower self-awareness, i.e., less ability to identify one’s own strengths and weaknesses, feelings, etc., which goes against an adequate self-perception and confidence in one’s own possibilities. These data are in complete contrast to the specific literature on the subject, as there is ample evidence of positive relationships between an individual’s level of physical activity and emotional dimensions [43,44,45,68].

### Limitations of the Study and Future Lines of Research

The limitations of the study include the absence of an experimental design, the impossibility of generalising results and the use of self-reports, among others. It would be desirable in future studies to use qualitative instruments, such as observation or interviews, in order to gain a deeper understanding of the characteristics that extracurricular activities should have in order to promote the acquisition of socioemotional skills. In particular, we have been particularly struck by the data relating to sporting activities; it would be necessary to analyse the quality of the programmes offered, the training of the professionals in charge of their development and the contents, methods and resources used. In this regard, the work of Durlak et al. [32] shows that when analysing the ASPs, only those that complied with the acronym SAFE (Sequenced, Active, Focused and Explicit) were effective. It also seems important to take into account the gender perspective in competence work, increasing self-management in girls and relationship management in boys.

## Figures and Tables

**Table 1:** Reliability by factors and scales.

	SECQ Score	SAW	SOAW	SM	RM	RDM	PAQ Score
Alfa of Cronbach	0.914	72	0.799	0.845	0.800	0.855	0.800
Number of elements	25	5	5	5	5	5	8

SAW: self-awareness; SOAW: social awareness; SM: self-management; RM: relationship management; RDM: responsible decision-making; SECQ Score: total score of the socio-emotional competences questionnaire; PAQ Score; total score of the Physical Activity questionnaire.

**Table 2:** Descriptors of the sample.

	Media	Md	Mo	SD	As	Curt	Min	Max
Weight (kg)	55.07	54	50	11.09	0.79	1.41	30	112
Height (cm)	163.5	163	160	10.13	−0.32	1.92	100	193
Age (years)	14.18	14	15	1.48	0.125	−0.83	11	18
BMI (kg/m2)	20.53	20.02	19.53	3.55	3.53	3.69	13.67	61.56
SECQ- Score	4.026	4	3.56	0.72	0.05	0.04	1	6
Self-awareness	4.47	4.4	5	0.78	−0.37	0.23	1	6
Social awareness	3.77	3.8	4	0.89	0.037	−0.344	1	6
Self-management	3.38	3.4	3	1.12	0.053	−0.578	1	6
Relationship management	4.42	4.4	4.4	0.912	−0.54	0.314	1	6
Responsible decision-making	4.06	4	4	1.00	−0.185	−0.34	1	6
PAQ-A Score	2.948	2.906	2.51	0.71	0.159	−0.268	1.13	5

Md: median; Mo: mode; SD: standard deviation; As: asymmetry; Curt: curtosis; Min: minimum; Max: maximum; BMI: Body Mass Index.

**Table 3:** Student’s t-test results for the sex variable in SECQ.

	Sex	N	M	SD	*t*	Sig
SECQ Score	Boy	466	4.04	0.751	0.832	0.406
	Girl	498	4.00	0.688		
Self-awareness	Boy	466	4.48	0.838		
	Girl	498	4.47	0.733	0.214	0.831
Social awareness	Boy	466	3.76	0.917		
	Girl	498	3.79	0.876	−0.538	0.591
Self-management	Boy	466	3.62	1.10		0.000
	Girl	498	3.15	1.10	6.604	
Relationship management	Boy	466	4.28	0.964		0.000
	Girl	498	4.56	0.837	6.604	
Responsible decision-making	Boy	466	4.07	0.966		
	Girl	498	4.04	1.04	−4.928	0.649

M: mean; SD: standard deviation; *t*: *t* value; Sig.: sigma.

**Table 4:** Student’s t test results for the variable attendance at extracurricular activities.

	Attendance	N	M	SD	*t*	Sig
SECQ Score	Yes	792	4.04	0.711	1.65	0.099
	No	172	3.94	0.750		
Self-awareness	Yes	792	4.4742	0.77322		
	No	172	4.4814	0.84321	−0.108	914
Social awareness	Yes	792	3.8051	0.89328		
	No	172	3.6430	0.90056	2.153	0.032
Self-management	Yes	792	3.3992	1.11618		
	No	172	3.3081	1.18259	0.960	0.337
Relationship management	Yes	792	4.4472	0.90143		
	No	172	4.3477	0.95895	1.298	0.195
Responsible decision-making	Yes	792	4.0912	0.98752		
	No	172	3.9372	1.09256	1.817	0.069

M: mean; SD: standard deviation; *t*: *t* value; Sig.: sigma.

**Table 5:** Analysis of variance (ANOVA) results for the relation between socio-emotional skills and type of extracurricular activities variables.

		N	Media	DS	*f*	Sig	Bonferroni
SECQ Score	None	153	3.94	0.748	4.369	0.002	None-Musical = 0.016 Musical-Sport = 0.011
	Musical	101	4.23	0.750			
	Sport	445	3.97	0.691			
	Academic	209	4.03	0.714			
	Artistic	56	4.23	0.734			
SAW	None	153	4.4732	0.84534	3.636	0.006	Musical-Sport = 0.008
	Musical	101	4.7030	0.75398			
	Sport	445	4.4121	0.77444			
	Academic	209	4.4545	0.76805			
	Artistic	56	4.6536	0.75268			
SOAW	None	153	3.6588	0.92449	3.341	0.010	None-Musical = 0.45 None-Artistic = 0.20
	Musical	101	3.9842	0.91025			
	Sport	445	3.7281	0.85679			
	Academic	209	3.8019	0.92943			
	Artistic	56	4.0071	0.90007			
SM	None	153	3.2837	1.19442	0.980	0.417	No differences
	Musical	101	3.4891	1.18169			
	Sport	445	3.4090	1.06607			
	Academic	209	3.3129	1.17779			
	Artistic	56	3.5179	1.13764			
RM	None	153	4.3765	0.94728	4.101	0.003	Musical-Sport = 0.018 Sport-Artistic = 0.043
	Musical	101	4.6634	1.01466			
	Sport	445	4.3501	0.87123			
	Academic	209	4.4469	0.91835			
	Artistic	56	4.7179	0.81064			
RDM	None	153	3.9320	1.09476	4.105	0.003	Musical-Sport = 0.018 None-Musical = 0.020
	Musical	101	4.3307	1.10397			
	Sport	445	3.9852	0.96322			
	Academic	209	4.1397	0.95917			
	Artistic	56	4.2821	1.00020			

SAW: Self-awareness; SOAW: Social awareness; SM: Self-management; RM: Relationship management; RDM: Responsible decision-makig.

**Table 6:** Correlations between the different factors and the independent variables.

		Age	PAQ Score	SAW	SOAW	SM	RM	RDM
**PAQ Score**	r	−0.198 **						
	Sig.	0.000						
SAW	r	−0.015	−0.081 *					
	Sig.	0.635	0.014					
SOAW	r	0.015	0.057	0.441 **				
	Sig.	0.632	0.083	0.000				
SM	r	0.063 *	0.054	0.425 **	0.395 **			
	Sig.	0.049	0.098	0.000	0.000			
RM	r	−0.009	−0.065	0.497 **	0.471 **	0.366 **		
	Sig.	0.780	0.059	0.000	0.000	0.000		
RDM	r	−0.045	0.023	0.563 **	0.500 **	0.501 **	0.574 **	
	Sig.	0.163	0.492	0.000	0.000	0.000	0.000	
SECQ Score	r	0.005	0.004	0.746 **	0.729 **	0.738 **	0.755 **	0.830 **
	Sig.	0.865	0.913	0.000	0.000	0.000	0.000	0.000

* *p* < 0.05; ** *p* < 0.001; SAW: Self-awareness; SOAW: Social awareness; SM: Self-management; RM: Relationship management; RDM: Responsible decision-making.

## Data Availability

The data are not publicly available due to confidentiality reasons.
